# Commentary on: Subpectoral Implant Repositioning With Partial Capsule Preservation: Treating the Long-Term Complications of Subglandular Breast Augmentation

**DOI:** 10.1093/asjof/ojab014

**Published:** 2021-04-26

**Authors:** Louis L Strock

**Affiliations:** Department of Plastic Surgery, University of Texas Southwestern Medical School, Dallas, TX, USA

Please watch a video commentary on “Subpectoral Implant Repositioning With Partial Capsule Preservation: Treating the Long-Term Complications of Subglandular Breast Augmentation” ^1^ featuring Dr Louis L. Strock, MD (Video).
